# Long-Term Cognitive Decline in MMA Fighters: A Two-Year Cohort Study on Executive Function Impairments Due to Repetitive Head Strikes

**DOI:** 10.3390/ijerph22071004

**Published:** 2025-06-26

**Authors:** Michele Andrade de Brito, José Raimundo Fernandes, Keveenrick Ferreira Costa, Clóvis Albuquerque Maurício, Aleksandro Ferreira Gonçalves, Otávio de Toledo Nóbrega, Esteban Aedo-Muñhoz, Ciro José Brito, Diego Ignácio Vanezuela Pérez, Bianca Miarka

**Affiliations:** 1 Graduate Program in Physical Education, Federal University of Rio de Janeiro, Rio de Janeiro 21941-617, Brazil; mybritto@hotmail.com (M.A.d.B.); clovisnutesportiva@gmail.com (C.A.M.); aleksandrofg@gmail.com (A.F.G.); miarkasport@hotmail.com (B.M.); 2 Universidade do Vale do Rio Doce, Governador Valadares 35020-220, Brazil; mundegv@hotmail.com (J.R.F.); keveenrick@hotmail.com (K.F.C.); 3 Graduate Program in Physical Education, Federal University of Juiz de Fora, Juiz de Fora 36036-900, Brazil; cirojbrito@gmail.com; 4 School of Physical Education, University of Brasilia, Brasilia 70910-900, Brazil; otavionobrega@unb.br; 5 Departamento de Educación Física, Deportes y Recreación, Universidad Metropolitana de Ciencias de la Educación, Ñuñoa 7760197, Chile; esteban.aedo@gmail.com; 6 Escuela de Kinesiologia, Universidad Santo Tomás, Santiago, Ñuñoa 7800262, Chile

**Keywords:** brain injuries, mixed martial arts, neuropsychological tests, athletic injuries, head trauma, memory disorders, executive function

## Abstract

Background: This study examines the long-term cognitive consequences of repetitive head impact on executive functions in mixed martial arts (MMA) fighters over two years. Methods: Neuropsychological tests assessed executive functions in two groups: competitive (CG; *n* = 26) and recreational groups (RG; *n* = 26). Results: A significant interaction between time and group was observed. The CG experienced greater declines in Mental Processing Speed (MPS) after 1 year [4.6 s (3.1; 6.1); *p* ≤ 0.001] and 2 years [14.6 s (5.1; 24.0); *p* = 0.003]. Inhibitory control (IC) also declined after 1 year [4.7 a.u. (3.0; 6.2); *p* ≤ 0.001] and 2 years [10.0 a.u. (11.6; 11.4); *p* ≤ 0.001]. Cognitive flexibility (CF) showed a pronounced reduction after 1 year [4.8 a.u. (3.0; 6.7); *p* ≤ 0.001] and 2 years [7.5 a.u. (5.9; 9.1); *p* ≤ 0.001]. Automatic (AP) and controlled processes (CP), as well as direct (DM) and indirect memory (IM), also showed significant impairments in CG. Conclusions: These findings highlight the detrimental effects of MMA competitions on cognitive function, emphasizing the need for monitoring and interventions to preserve fighters’ health and performance.

## 1. Introduction

Concussions refer to brain damage caused by a sudden impact on the head, such as a blow or rapid movement that forces the brain to displace within the cranial cavity, leading to changes in brain function [1,2]. In combat sports, particularly among mixed martial arts (MMA) practitioners, the risk of concussion is frequent due to the nature of combat, which combines high intensity grappling and striking techniques [3]. This situation has raised significant concerns [4,5], given that the prevalence, intensity, and severity of concussions are particularly high in this sport [3,6,7,8]. Previous studies highlight that these injuries can have short- and long-term effects on cognitive function and neurochemical balance, ultimately impairing an athlete’s performance and quality of life [4,7,8,9,10]. These impacts are linked to physical and functional changes in the brain, including neurodegeneration, chronic inflammation, and diffuse axonal damage, which can progress to conditions such as chronic traumatic encephalopathy and progressive cognitive decline [11,12,13,14].

Due to the frequency of head injuries in MMA, it is important to investigate their long-term impacts to develop effective prevention and rehabilitation strategies [8]. Distinguishing between single impacts, which result in acute concussion, and repetitive subconcussive impacts, which do not trigger immediate symptoms but accumulate over time, is critical to understanding long-term risks [15,16]. Concussion manifests obvious deficits, and subconcussive traumas are silent but insidious [17]. Studies among American football, boxing, and rugby athletes demonstrate that these repetitive impacts are associated with gradual reductions in the volume of brain regions such as the hippocampus and prefrontal cortex, as well as impairments in executive functions such as planning, working memory, and inhibitory control [15,18,19,20].

In this context, assessing executive functions becomes a necessary tool for the early detection of cognitive deficits. This assessment measures skills such as cognitive flexibility (switching between combat modalities and adjusting tactics according to the dynamic context of the fight), processing speed (decoding complex stimuli and transforming them into efficient motor responses essential for defense and counterattacks), the inhibition of automatic responses (avoiding impulsive responses by maintaining focus on strategic actions even under physical and emotional pressure) and working memory (managing and accessing strategic knowledge and past experiences to make decisions) [8,21,22,23]. In addition to being non-invasive, it is easy to apply and offers insights into the integrity of the prefrontal cortex, which is a region vulnerable to traumatic injuries. Its use in public health allows the identification of cognitive deficits, allowing early interventions and reducing costs associated with long-term treatments, such as cognitive rehabilitation, psychological support, and palliative care for degenerative conditions [21,22,24,25].

Given the critical need to monitor cognitive decline in MMA fighters, this study aims to investigate the relationship between repetitive head blows and executive impairment in MMA fighters, not only to expand our understanding of neuropsychological risks in this sport but also to support prevention policies, safe return to activities protocols, and awareness campaigns. This two-year cohort study thus seeks to analyze the trajectories of cognitive decline in this population, using the assessment of executive functions as a central marker, contributing to an urgent debate on sports safety and long-term brain health.

## 2. Materials and Methods

### 2.1. Study Design

This study employed a prospective, two-year cohort design to assess executive functions in MMA athletes exposed to recurrent head impacts compared to athletes who train recreationally without sparring or competition participation. The research was approved by the Research Ethics Committee of the Federal University of Rio de Janeiro (protocol 48167021.5.0000.5257) and conducted in accordance with the Declaration of Helsinki. Following this approval, two MMA training centers were contacted, where the study objectives, potential risks, and voluntary nature of participation were thoroughly explained. Participants who met the inclusion criteria and consented to take part signed the Informed Consent Form.

**Figure 1:**
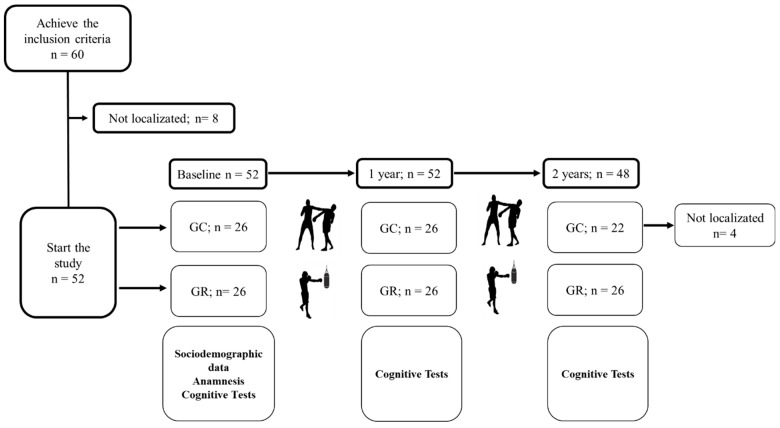
Study design, organized into participant selection and timeline: competitive group (CG) and recreational group (RG).

Participants were divided into two paired groups based on their exposure to head impacts: competitive and recreational. All participants were required to have a minimum of five years of MMA experience to ensure a comparable baseline skill level. To ensure reliability, training sessions were systematically recorded and analyzed to quantify head strike exposure. The number of head impacts per session was self-reported by the athletes and cross-referenced with video footage reviewed by independent raters. Potential confounders, such as age, training frequency, official fights, sparring, and baseline cognitive performance, were controlled during data analysis. Cognitive testing was performed immediately pre- and post-sparring, with follow-up after 1 year and 2 years. Cognitive evaluations were conducted by a licensed neuropsychologist under standardized conditions. Neuropsychological assessments were performed at three time points: baseline, one year, and two years, as illustrated in Figure 1.

### 2.2. Participants’ Characteristics and Baseline Measures

To achieve the study objectives, MMA practitioners were selected and divided into two groups based on specific inclusion criteria. The competitive group (CG) comprised athletes regularly exposed to high-impact training, while the recreational group (RG) included practitioners with minimal head strike exposure.

For the CG, the inclusion criteria were as follows: (a) age ≥ 18 years; (b) at least five years of MMA practice; (c) training sessions lasting ≥60 min, at least three times per week; (d) regular participation in official competitions; (e) at least two weekly sparring sessions involving head strikes; (f) sparring without protective headgear; (g) no history of diagnosed concussion within 60 days prior to the study.

For the RG, the same criteria were applied except for subitems (d) and (e), which were replaced with the following: (a) engagement in MMA training without sparring or head-impact drills; (b) no participation in MMA competitions.

The exclusion criteria for both groups included the following: (a) a history of neurological or psychiatric disorders and (b) incomplete participation in all stages of the protocol.

During the initial contact with the selected athletes, an anamnesis and sociodemographic questionnaire were administered to collect information on body mass (kg), height (cm), age (years), practice time (months), training duration (minutes), and, for CG, the number of sparring sessions per week [26]. Training sessions were randomly recorded, and data were cross-checked with the information provided by the athletes [27]. We used the FDT cutoff points based on the standards of refs [28,29] and stratified by age and education.

### 2.3. Training Structure and Head Impact Exposure

For this study, two groups were organized based on the inclusion criteria. Table 1 outlines a typical training week for the CG and RG.

The training sessions for both groups ranged from 90 to 120 min. All sessions included 8–15 min of warm-up and a 5 min cool-down period at the end.

Striking sessions focused on technical drills and skill development in Muay Thai, incorporating punches, kicks, elbows, and knee strikes in a controlled environment with specific striking equipment such as focus mitts, kick pads, and punching bags [13].

Grappling sessions emphasized wrestling and Brazilian Jiu-Jitsu techniques, including takedowns, positional control, submissions, and escape [30].

MMA sessions integrated elements of both striking and grappling, allowing participants to transition between standing and ground combat scenarios.

Sparring sessions were structured to mimic competition conditions, consisting of two or three combat simulations. Each simulation comprised three rounds, with each round lasting three minutes interspersed with a one-minute recovery interval [31].

To quantify head impact exposure, sparring sessions were video-recorded (Smartphone Samsung Galaxy S21, SAMSUNG, South Korea), and independent raters reviewed the footage to count the number of head strikes sustained per session [3].

### 2.4. Cognitive Measures

To assess executive functions, two validated neuropsychological tests were selected:

The Five-Digit Span Test (FDT): This evaluates executive functions through indices of Mental Processing Speed (MPS), inhibitory control (IC), cognitive flexibility (CF), and automatic (AP) and controlled processes (CP) by presenting conflicting numerical information. Longer completion times indicate lower performance [10,31].

The Digit Span Test: This measures working memory capacity by assessing direct memory (DM) and indirect memory (IM). The indirect order is more complex, requiring short-term storage and information processing, while the direct order requires only the passive storage of verbal material. Performance is scored based on the total number of recalled digits, with fewer recalled digits indicating worse cognitive performance [32,33].

### 2.5. Statistical Analysis

Data were initially organized into contingency tables for baseline, one-year, and two-year assessments, with comparisons made between the CG and RG. Descriptive statistics were reported as means ± standard deviations (SDs) or medians with interquartile ranges (IQRs), depending on data distribution. Categorical variables were expressed as frequencies and percentages. Normality of the data was assessed using the Shapiro–Wilk test [31].

GEEs were employed to analyze within-group and between-group differences over time, accounting for correlated observations and intra-subject variability [34,35]. An exchangeable correlation structure was applied, and model selection was based on the Quasi-Likelihood under the Independence Model Criterion for optimal model fit. Bonferroni post hoc pairwise comparisons were used for multiple comparisons.

Effect sizes (ESs)—Cohen’s d′ for continuous variables and Cramer’s V for categorical data—were calculated to quantify the magnitude of observed differences. Cohen’s d′ values were used to interpret the values as follows: 0.2 (small), 0.5 (medium), and 0.8 (large); Cramer’s V was used as follows: (0.0) no association, (0.1) small, (0.3) medium, and (0.5) large. n2 was (0.01) small; (0.06) medium; or (0.14) large [34]. Statistical analyses were performed using SPSS version 25.0 (IBM, Chicago, IL, USA), with significance set at *p* ≤ 0.05 [36].

## 3. Results

The analysis focused on the differentiation between recreational and competitive athletes, highlighting the consequences of repetitive impacts on the executive functions and cognition of athletes. A difference was observed in the characterization of the groups, as demonstrated in Table 2.

Significant differences were found between the groups for age, body mass, training sessions per week, and the number of head impacts received by blows (i.e., kicks, knees, punches, and elbows).

**Figure 2:**
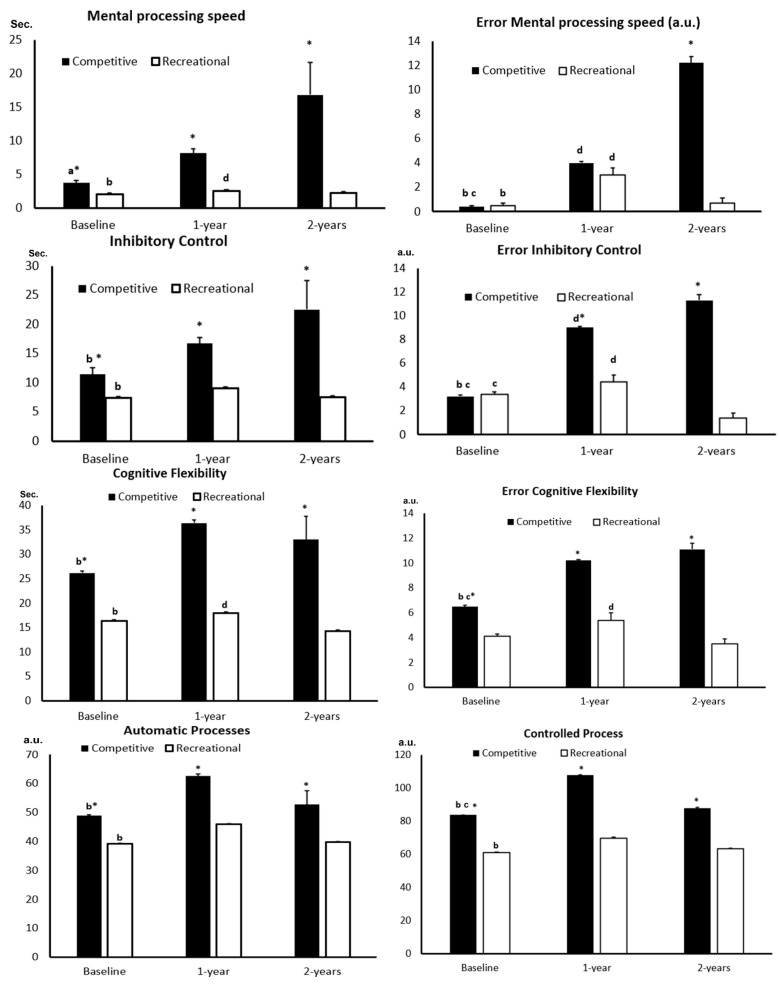

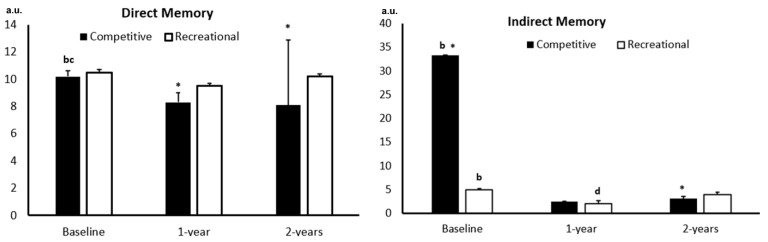
Analysis of executive functions of the groups. Legend: ^a^ *p* ≤ 0.02 baseline vs. 1 and 2 years; ^b^ *p* ≤ 0.001 baseline vs. 1 year; ^c^ *p* ≤ 0.001 baseline vs. 2 years; ^d^ *p* ≤ 0.001 at time point 1 year vs. 2 years; * *p* ≤ 0.003 versus recreational for this time point; a.u. arbitrary units.

For the current group of participants, no test had to be repeated at any stage, as there were no incidents that affected performance during the administration of the instruments. Based on the data presented in Table 1, it is evident that the CG presented a higher exposure to head impacts, which could influence the variables measured in this study. Figure 2 presents the results for the MPS, IC, CF, AP, CP, DM, and IM tests, along with the associated errors for each measurement.

### 3.1. Mental Processing Speed (MPS)

A time × group interaction effect was observed (W = 19.8; df = 2; *p* ≤ 0.001). The CG showed a significant difference between baseline and 1 year [−4.4 s (−6.1; −2.7); *p* ≤ 0.001; d′ = 2.6], as well as between baseline and 2 years [−13.1 s (−24.6; −1.5); *p* = 0.02; d′ = 1.3]. The RG also exhibited significant differences between baseline and 1 year [−1.5 s (−2.0; −1.0); *p* ≤ 0.001; d′ = 3.0] and between 1 year and 2 years [−1.2 s (−0.5; −2.0); *p* ≤ 0.001; d′ = 1.6]. When comparing the groups, significant differences were found at all time points: baseline {[1.7 s (0.7; 2.7); *p* = 0.001]}, 1 year {[4.6 s (3.1; 6.1); *p* ≤ 0.001]}, and 2 years {[14.6 s (5.1; 24.0); *p* = 0.003; d′ = 1.7]}. Regarding MPS errors, a time × group interaction effect was also observed (W = 392.4; df = 2; *p* ≤ 0.001). The CG showed significant differences between baseline and 1 year [−3.6 a.u. (−5.9; −1.3); *p* = 0.001; 3.1], baseline and 2 years [−11.8 a.u. (−13.1; −10.5); *p* ≤ 0.001; d′ = 1.5], and between 1 year and 2 years [−8.2 a.u. (−10.5; −5.9); *p* ≤ 0.001]. The RG showed significant differences between baseline and 1 year [−2.5 a.u. (−3.9; −1.1); *p* = 0.001] and between 1 year and 2 years [−2.3 a.u. (−0.9; −3.8); *p* = 0.001]. Group comparisons revealed a significant difference only at the 2-year time point [11.5 a.u. (10.4; 12.6); *p* ≤ 0.001].

### 3.2. Inhibitory Control (IC)

A significant time × group interaction effect was observed (W = 35.5; df = 2; *p* ≤ 0.001). The CG showed a significant difference between baseline and 1 year [5.3 s (4.0; 6.8); *p* ≤ 0.001; d′ = 3.8]. The RG also showed a difference between baseline and 1 year [1.6 s (0.9; 2.4); *p* ≤ 0.001; d′ = 2.1]. Comparisons between groups revealed significant differences at all time points as follows: baseline {[4.0 s (1.4; 6.5); *p* = 0.002; d= 1.6]}, 1 year {[7.7 s (5.6; 9.8); *p* = 0.001; d′ = 3.6]}, and 2 years {[15.0 s (5.1; 25.0); *p* = 0.003; d′ = 1.5]}. Regarding CI errors, a significant time × group interaction effect was also observed (W = 99.9; df = 2; *p* ≤ 0.001). The CG demonstrated significant differences between baseline and 1 year [5.8 a.u. (4.4; 7.2); *p* ≤ 0.001], baseline and 2 years [8.1 a.u. (6.2; 10.0); *p* ≤ 0.001], and between 1 year and 2 years [2.3 a.u. (0.7; 3.9); *p* = 0.002]. The RG showed differences between baseline and 2 years [2.1 a.u. (0.5; 3.7); *p* = 0.006] and between 1 year and 2 years [3.0 a.u. (1.4; 4.6); *p* ≤ 0.001]. Group comparisons revealed significant differences at the 1-year {[4.7 a.u. (3.0; 6.2); *p* ≤ 0.001]} and 2-year {[10.0 a.u. (11.6; 11.4); *p* ≤ 0.001]} time points.

### 3.3. Cognitive Flexibility (CF)

A time × group interaction effect was identified (W = 16.0; df = 2; *p* ≤ 0.001). The CG showed a significant difference between baseline and 1 year [10.2 s (3.7; 16.6); *p* ≤ 0.001; d′ = 1.6], while the RG showed a difference between baseline and 1 year [1.6 s (1.0; 2.3); *p* ≤ 0.001; d′ = 2.5]. The RG did not show significant differences beyond this. Comparisons between groups revealed differences at all time points as follows: baseline {[9.8 s (5.8; 13.8); *p* ≤ 0.001; d′ = 2.5]}, 1 year {[18.4 s (11.6; 25.1); *p* ≤ 0.001; d′ = 2.7]}, and 2 years {[18.7 s (10.2; 27.2); *p* ≤ 0.001; d′ = 2.2]}. For CF errors, the time × group interaction effect was also significant (W = 12.5; df = 2; *p* = 0.002). The CG showed significant differences between baseline and 1 year [3.7 a.u. (1.7; 5.7); *p* ≤ 0.001] and between baseline and 2 years [4.2 a.u. (2.0; 7.2); *p* ≤ 0.001]. Group comparisons revealed significant differences at all time points as follows: baseline [2.3 a.u. (0.2; 4.5); *p* = 0.03], 1 year [4.8 a.u. (3.0; 6.7); *p* ≤ 0.001], and 2 years [7.5 a.u. (5.9; 9.1); *p* ≤ 0.001].

### 3.4. Automatic Processes (AP)

A time × group interaction effect was found (W = 17.4; df = 2; *p* ≤ 0.001). The CG showed significant differences between baseline and 1 year [13.7 a.u. (9.9; 17.6); *p* ≤ 0.001; d′ = 3.6], while the RG showed differences between baseline and 1 year [6.8 a.u. (5.1; 8.5); *p* ≤ 0.001; d′ = 4.0] and between 1 year and 2 years [6.2 a.u. (2.8; 9.6); *p* ≤ 0.001; d′ = 1.8]. Comparisons between groups revealed significant differences at all time points as follows: baseline {[9.8 a.u. (5.0; 14.6); *p* ≤ 0.001; d′ = 2.0]}, 1 year {[16.7 a.u. (11.2; 22.2); *p* ≤ 0.001; d′ = 3.0]}, and 2 years {[13.1 a.u. (4.8; 21.4); *p* = 0.002; d′ = 1.6]}.

### 3.5. Controlled Processes (CP)

Another interaction effect between group and time was observed (W = 23.8; df = 2; *p* ≤ 0.001). The CG showed significant differences between baseline and 1 year [24.1 a.u. (16.6; 31.6); *p* ≤ 0.001; d′ = 3.2] and between baseline and 2 years [19.9 a.u. (4.1; 35.7); *p* = 0.008; d′ = 1.3]. The RG showed a significant difference only between baseline and 1 year [8.6 a.u. (6.9; 10.3); *p* ≤ 0.001; d′ = 5.1]. Comparisons between groups revealed significant differences at all time points as follows: baseline {[22.6 a.u. (15.0; 30.3); *p* ≤ 0.001; d′ = 3.0]}, 1 year {[38.1 a.u. (29.2; 47.0); *p* ≤ 0.001; d′ = 4.3; d′ = 2.3]}, and 2 years {[24.9 a.u. (13.9; 35.9); *p* ≤ 0.001]}

### 3.6. Direct Memory (DM) and Indirect Memory (IM)

For direct memory, a significant interaction effect between group and time was observed (W = 5.8; df = 2; *p* = 0.05). The CG showed significant differences between baseline and 1 year [1.8 a.u. (1.4; 2.3); *p* ≤ 0.001; d′ = 4.0] and between baseline and 2 years [2.1 a.u. (1.2; 2.9); *p* ≤ 0.001; d′ = 2.5]. No significant differences were observed in the RG. Group comparisons revealed significant differences at both 1 year [1.2 a.u. (0.5; 1.9); *p* ≤ 0.001; d′ = 1.7] and 2 years [2.1 a.u. (1.2; 2.9); *p* ≤ 0.001; d′ = 2.5]. For indirect memory, a significant interaction effect between group and time was also observed (W = 19.7; df = 2; *p* ≤ 0.001). The CG showed significant differences between baseline and 1 year [0.9 a.u. (0.1; 1.6); *p* = 0.01; d′ = 1.2]. The RG showed significant differences between baseline and 1 year [2.9 a.u. (2.0; 3.9); *p* ≤ 0.001; d′ = 3.1] and between 1 year and 2 years [1.9 a.u. (1.1; 2.8); *p* ≤ 0.001; d′ = 2.2]. Group comparisons revealed significant differences at baseline [1.7 a.u. (0.7; 2.6); *p* = 0.001; d′ = 1.8] and at 2 years [0.9 a.u. (0.2; 1.6); *p* = 0.008; d′ = 1.3].

## 4. Discussion

Sports practice presents a high risk for concussion, with brain injuries being among the most common, often occurring repeatedly in the same athlete [26,27]. MMA athletes face an increased exposure to such risks due to the nature of the sport [13]. To estimate the long-term damage caused by concussions, it is essential to apply neuropsychological tests, which serve as crucial tools for assessing, monitoring, and tracking executive function impairments throughout MMA training. These functions are fundamental for both sports performance and overall quality of life [2]. Neuropsychological tests not only facilitate the early detection of cognitive deficits resulting from concussions but also allow for the implementation of targeted interventions to mitigate long-term damage [8].

Given this context, the present study aimed to estimate the long-term cognitive impacts on MMA practitioners who spar and compete compared to those who only practice the sport recreationally. Using a longitudinal paired-cohort protocol, baseline cognitive measures were collected and reassessed after one and two years. The primary findings of this study indicate progressive impairments across all cognitive measures in the CG at both follow-up points, except for the MPS error and IM, where significant differences emerged only after two years (*p* ≤ 0.003 versus RG for this measurement time point).

Chronic exposure to repeated head trauma during sparring is a critical factor that contributes to a progressive executive function decline in combat sports athletes [32,37]. Sparring, which closely mimics real combat, frequently involves repeated head impacts, leading to cumulative neurological damage [13,23,38]. These findings align with prior research, which demonstrated that these injuries lead to lasting neuropsychological deficits, impairing decision-making, focus, and impulse control in athletes [3,23,29,39]. The detrimental effects of sparring on executive functions extend beyond sports performance, as these cognitive skills are fundamental for daily life and overall well-being [32,35]. Therefore, strategies aimed at mitigating the cognitive impact of concussions should be prioritized, addressing both athletic performance and long-term neurological health [8].

At baseline, the CG already exhibited deficits in MPS, IC, CF, CF error, AP, CP, and IM, likely due to their long-term exposure to sparring and competitive training. However, the most concerning aspect was that, after one year, cognitive impairments persisted and worsened across all measured variables except for MPS error and IM. By the second year, CG participants showed significant impairments in all cognitive domains relative to the RG. These findings underscore the importance of implementing routine neuropsychological monitoring for combat sports athletes to track cognitive deterioration over time. Even when neuroimaging does not detect structural injuries, neuropsychological evaluation remains crucial for identifying functional cognitive impairments, predicting long-term prognosis, and diagnosing potential neurodegenerative conditions at an early stage [33].

The increased reaction time and MPS errors observed in the CG suggest that repeated exposure to sparring elevates the likelihood of concussion. As shown in Table 1, the CG had more training sessions than the RG; however, the primary difference lies in the significantly greater number of head impacts sustained by CG participants. These findings contrast with acute studies that identified immediate executive function impairments following sparring without head protection [1,32]. MPS reflects the brain’s ability to process and respond to stimuli quickly, which is a crucial ability in fast-paced combat situations where milliseconds can determine the outcome of a fight. Impairments in this function not only decrease athletic performance but also increase the risk of further concussions due to delayed reaction times and impaired coordination.

The IC results followed a similar pattern to MPS, reinforcing the notion that executive dysfunction contributes to a greater risk of poor decision-making and decreased tactical efficiency. This places athletes at a disadvantage in competitive settings and increases their susceptibility to further injuries [21]. These findings align with prior research reporting a progressive decline in IC among MMA and boxing athletes over time [22]. Inhibitory control is a critical executive function that enables athletes to suppress impulsive reactions and execute well-planned strategies under pressure, as demonstrated in prior studies [29].

Regarding CF and CF errors, the chronic negative effect of competitive MMA practice was particularly evident. The progressive decline observed over the two-year period suggests that the cumulative effects of concussions contributed to these impairments, reinforcing the findings of this longitudinal study. Acute research has shown that a single sparring session results in immediate CF impairment, negatively affecting an individual’s ability to switch between the different executive tasks necessary for adjusting strategies during combat [1]. The chronic nature of these impairments highlights the need for the ongoing monitoring of executive functions in MMA athletes.

The observed deficits in AP and CP indicate impairments in high-order executive skills, including planning, decision-making, and conscious control of actions [10,21]. These results suggest that repeated exposure to head impacts significantly affects cognitive performance, potentially compromising reaction times and the ability to execute complex motor sequences. Notably, this study is among the first to evaluate these specific measures in combat sports, as limited scientific evidence exists regarding the assessment of automatic and controlled processes in MMA. Future research should further explore these cognitive domains to strengthen the understanding of their impact on athletic performance and injury risk. The inability to perform automatic and controlled tasks efficiently may hinder an athlete’s ability to execute fundamental movements with precision, increasing the risk of technical errors and injuries [10].

Regarding DM and IM, impairments in these functions could disrupt cognitive processing during training and competition, negatively affecting an athlete’s ability to integrate tactical instructions, anticipate opponents’ movements, and execute strategies under pressure [1,32]. These findings align with prior research indicating that exposure to concussions causes significant long-term memory impairments [8]. However, previous studies have primarily assessed memory function as a broad category rather than focusing on specific subdomains such as DM and IM [3,24,35].

The idea that the cessation of fighting practice in MMA athletes could lead to the spontaneous improvement of cognitive impairment deserves critical consideration. Contrary to this expectation, evidence from high-impact sports such as boxing and American football suggests that injuries resulting from repetitive trauma are associated with irreversible neurodegenerative processes characterized by tau protein accumulation and diffuse axonal loss [16,40]. In our study, the observed deficits in executive functions and processing speed may reflect similar cumulative damage, which, once established, tends to stabilize or progress, even in the absence of new impacts. Although early retirement may prevent worsening, functional recovery would require active neuropsychological rehabilitation interventions, as observed in populations with chronic traumatic brain injury [16]. These findings reinforce the need for continuous cognitive monitoring protocols and early stimulation programs for MMA athletes, mitigating the risks of dementia associated with high-impact sports [8,16,41,42].

Despite its significant contributions, this study has several limitations. One major limitation is the scarcity of longitudinal studies examining the chronic cognitive effects of MMA participation, making direct comparisons with previous research challenging. Most existing studies have focused on acute effects, systematic reviews, or epidemiological investigations [3,7,10,38]. This gap underscores the need for more long-term research in combat sports to fully understand the progressive impact of repetitive head trauma on executive functions and memory. Another important limitation is the heterogeneity of cognitive assessment tools across studies, which complicates direct comparisons between findings. The lack of a standardized neuropsychological evaluation protocol for combat sports introduces variability in how cognitive impairments and concussion effects are measured. Future research should aim to establish a universally accepted set of neuropsychological tests tailored to the specific demands of MMA and other high-impact sports.

### Practical Applications

The findings of this study suggest that neuropsychological tests used in this research should be integrated into routine assessments for MMA fighters, as they provide an effective means of monitoring potential cognitive decline [24,39,43]. These assessments are quick to administer, minimally disruptive to training schedules, and suitable for periodic application [24,39,43]. The resulting data should be analyzed by the athlete’s support team—including coaches, physicians, and psychologists—to develop cognitive rehabilitation programs and preventive strategies [24,39,43]. These interventions should be incorporated alongside technical and tactical training to mitigate cognitive impairments and facilitate executive function recovery, ensuring athletes can compete safely and maintain optimal performance. In extreme cases, this information may even assist in decision-making regarding an athlete’s retirement. Additionally, athletes, coaches, and support staff should be educated on the risks of concussions and their potential cognitive consequences. We recommend workshops and lectures to enhance awareness of concussion symptoms, emphasizing the importance of neuropsychological monitoring and highlighting the long-term benefits of proactive cognitive care.

## 5. Conclusions

This study provides compelling evidence that MMA athletes exposed to frequent sparring experience significant and progressive impairments in executive functions, including Mental Processing Speed, inhibitory control, cognitive flexibility, automatic processes, controlled processes, direct memory, and indirect memory. These findings highlight the cumulative impact of repetitive head trauma on cognitive performance, reinforcing the need for systematic neuropsychological monitoring in combat sports.

Given the long-term neurological risks associated with repeated head impacts, the integration of standardized cognitive assessments into routine evaluations for MMA athletes is critical. This study underscores the urgency of establishing standardized cognitive assessment protocols and prioritizing athlete safety in combat sports.

## Figures and Tables

**Table 1:** Typical week of training for competitive and recreational MMA groups.

Monday	Tuesday	Wednesday	Thursday	Friday	Saturday
Competitive group morning					
Grappling (≈120 min)	Sparring (≈60 min)	Grappling (≈120 min)	Sparring (≈60 min)	Grappling (≈120 min)	MMA (≈120 min)
Competitive group evening					
Striking (≈120 min)	-	Striking (≈120 min)	-	Striking (≈120 min)	-
Recreative group evening					
Striking (≈120 min)	Grappling (≈120 min)	Striking (≈120 min)	Grappling (≈120 min)	Striking (≈120 min)	MMA (≈120 min)

Note: MMA—mixed martial arts.

**Table 2:** Comparison between MMA competitive and recreational groups under baseline conditions.

Measure	CG	RG	Statistics (T_calc_.; *p*; CI 95%)	ES
Age (yrs.)	26.7 ± 3.7 *	32.0 ± 8.2	T = −2.99; *p* = 0.004; −5.3 (−8.8; −1.7)	0.8
Weight (kg)	72.0 ± 8.4 *	78.7 ± 8.2	T = −2.92; *p* = 0.005; −6.7 (−11.4; −2.1)	0.8
Height (cm)	170.0 ± 0.1	1.7 ± 0.1	T = −0.149; *p* = 0.88; −0.0 (−0.0; 0.0)	0.1
Practice time (months)	47.8 ± 5.2	46.9 ± 5.0	T = −0.665; *p* = 0.5; 0.9 (−1.8; 3.7)	0.2
Sessions per week	9.7 ± 2.5 *	4.7 ± 1.1	T = 9.26; *p* ≤ 0.001; 5.0 (3.9; 6.1)	2.6
Training duration (min)	101.5 ± 28.2	97.2 ± 28.5	T = 0.55; *p* = 0.58; 4.3 (−11.5; 20.1)	0.2
Blows received to the head/ses.	15.0 ± 1.6 *	2.4 ± 1.4 ^#^	T = 30.6; *p* ≤ 0.001; 12.6 (11.8; 13.4)	8.4
Sleep (hours)	7.4 ± 1.0 *	6.6 ± 1.0	T = 3.09; *p* = 0.003; 0.9 (0.3; 1.4)	0.8

^#^ Accidental training blows; min—minutes; ses—session. Tcalc—T calculated. * *p* ≤ 0.005. CG—control group; RG—recreational group; ES—effect size.

## Data Availability

The original contributions presented in this study are included in the article. Further inquiries can be directed to the corresponding authors.
